# Delayed Esophageal Pseudodiverticulum after Anterior Cervical Spine Fixation: Report of 2 Cases 

**Published:** 2015-03

**Authors:** Ali Sadrizadeh, Ehsan Soltani, Mehdi Abili, Paria Dehghanian

**Affiliations:** 1 *Department of Thoracic Surgery, Ghaem Hospital, School of Medicine, Mashhad University of Medical Sciences, Mashhad, Iran.*; 2 *Acute Care Surgery Research Center, Taleghani Hospital, School of Medicine, Mashhad University of Medical Sciences, Mashhad, Iran.*

**Keywords:** Anterior cervical spine fixation, Conservative management, Delayed postoperative complication, Esophageal perforation, Esophageal diverticulum, Fistula management, Plate fixation

## Abstract

**Introduction:**

Although perforation of the esophagus, in the anterior cervical spine fixation, is well established, cases with delayed onset, especially cases that present pseudodiverticulum, are not common. In addition, management of the perforation in this situation is debated.

**Case Report:**

Delayed esophageal pseudodiverticulum was managed in two patients with a history of anterior spine fixation. Patients were operated on, the loose plate and screws were extracted, the wall of the diverticulum was excised, the perforation on the nasogastric tube was suboptimally repaired, and a closed suction drain was placed there. The NGT was removed on the 7th day and barium swallow demonstrated no leakage at the operation site; therefore, oral feeding was started without any problem

**Conclusion:**

In cases with delayed perforation, fistula, or diverticulum removal of anterior fixation instruments, gentle repair of the esophageal wall without persistence on definitive and optimal perforation closure, wide local drainage, early enteral nutrition via NGT, and antibiotic prescription is suggested.

## Introduction

Nowadays, anterior cervical spine fixation is a popular method. In the last two decades, since Robinson proposed this method for spine fixation, a large number of advancements have been made (1,2). The original technique was based on plate and screw fixation, with or without bone implants, or grafting. Recently neurosurgeons and spine surgeons used new instruments that are highly tolerated by the body, and do not need to be extracted. 

This method is useful in spinal disorders such as trauma, spondylosis, metastatic disease, spine deformity, and degenerative diseases. Esophageal perforation or fistula is an uncommon but well-recognized complication of the procedure. This complication can be seen during interoperation, post operation, or in a later period. Here, the rarity of the esophageal pseudodiverticulum complication is not only discussed; but also the limitation of related reports is examined. 

***Case 1***

A 46-year-old woman underwent anterior cervical spine fixation, 7 years ago, due to neck trauma. She complained postprandial chest pain for 8 months. Moreover, for 3 months, she had intermittent dysphagia to solid materials, in addition to halitosis.

**Fig 1:**
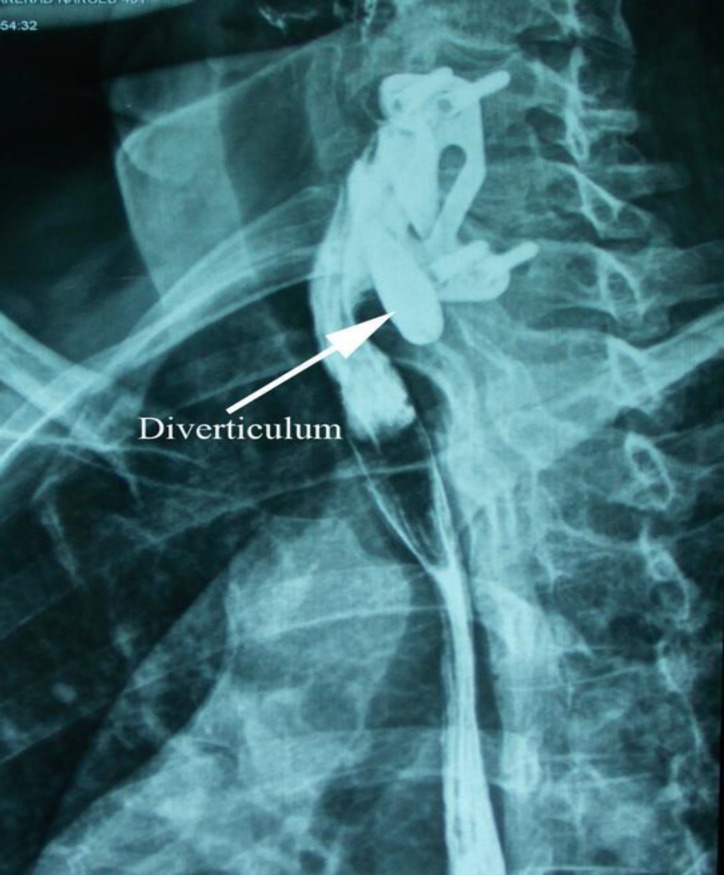
Esophageal pseudodiverticulum near the plate fixation site in case one

First, we performed a barium swallow study that showed an esophageal pseudodiverticulum near the plate fixation site (Fig.1). An upper endoscopy was requested that confirmed the posterior pseudodiverticulum without any dysplasia in the pathology result.

After a neurosurgery consultation, the patient was operated on. She had a pseudodiverticulum with severe inflammation, fibrosis, and adhesion to the plate. The screws were very loose, and the plate was extracted easily. Due to the complete fusion of the spine, attempts to fix it again were not undertaken. The wall of the pseudodiverticulum was excised, but due to severe inflammation, the wall couldn’t be repaired optimally. Since inflammation and fibrosis were observed in the entirety of the soft tissue around the diverticulum, and damage to important structures in the neck was a concern, only a closed suction drain was placed and the operation was terminated. A muscle flap was used to cover the esophageal defect. A nasogastric tube (NGT) was placed intraoperatively and feeding was started on the first postoperative day. Three days after surgery, there was a little drainage and the drain was extracted. The NGT was removed on the 7th day and barium swallow demonstrated no leakage at the operation site, so oral feeding was started without any problem. 3 months later, a barium study was requested which showed no stricture or other problems.

***Case 2***

A 24-year-old man, who suffered from quadriplegia, following spinal cord injury, and from a fracture of C5 and C6 due to trauma, underwent anterior cervical spine fixation with plate, screw, and posterior fixation rods 2.5 years ago. At this time, he had been suffering from odynophagia and intermittent dysphagia for 2 months.

**Fig 2:**
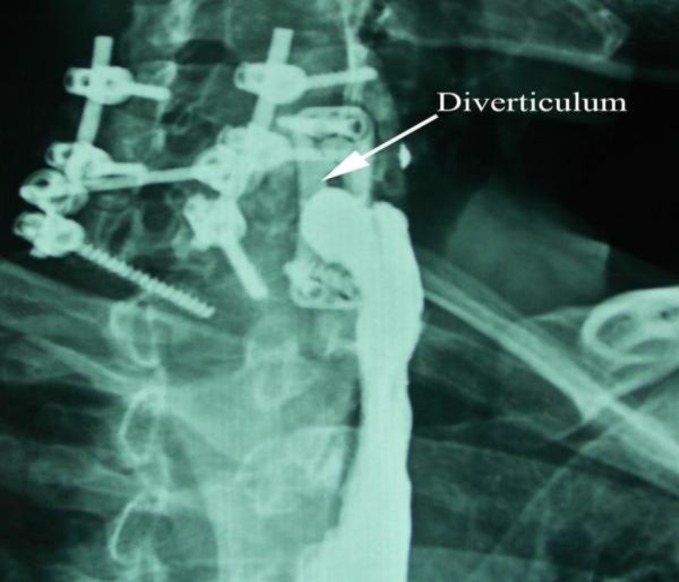
Esophageal pseudodiverticulum near the plate fixation site in case two

Moreover, barium study revealed a posterior pseudodiverticulum at the site of spinal fixation (Fig.2). Intraoperative findings were similar to case one and our approach was similar as well. The outcome was positive.

## Discussion

The fixation of the cervical spine via the anterior approach is an effective and well-recognized procedure, which was used for numerous problems such as spondylotic disease, cervical myelopathy, tumors, and trauma (1,2-5). Several specific complications were reported for this procedure and the esophageal perforation or fistula is one of the most uncommon but well-recognized complications.

Although the perforation of the esophagus in this procedure is uncommon, it is well established (1,5-9). Its incidence is estimated between 0.02% and 1.49% (12-14). It may be further subdivided into acute or delayed injuries. One of the most common causes of acute injuries is careless usage of fixation instruments (1,10,12,18). In this situation, the patients complain about pain in the neck, odynophagia, and dysphagia, and fever, neck erythema, neck incision discharge, and leukocytosis are observed (1,9,19). The treatment of this complication is well established in the literatures; and therefore, is not discussed further.

Delayed injuries are due to plate compression, erosion, and the microtrauma effect on the posterior wall of the esophagus (1,2,12,21). The result of this effect is screw, and in rare cases, plate migration into the gastrointestinal tract, which may be completely asymptomatic (2,4,10,21). 

Sometimes esophageal compression and chronic friction by internal fixators can develop an adhesive-traction pseudodiver- ticulum (1,9,21). Clinical presentations are similar to esophageal diverticulum. In contrast, pharyngo-esophagial study can confirm the diagnosis. Cervical spine X-Ray imaging and neck CT scan should be performed to evaluate an abscess formation and or plate and screw displacement (3,12,18).

Despite true diverticulum, in these cases, surgeons are faced with a pseudodiver- ticulum and severe inflammation and fibrosis in the surrounding tissue (11). The previous neck operation worsens the situation for the surgeon. Moreover, plating plays a foreign body role and hope for improvement is poor if the preservation of the location is insisted upon. The classic treatment, in these cases, is removal of anterior fixation instruments and reparation of the defect with a vascularized muscle flap (1,11,12). 

So in cases with delayed perforation, fistula or pseudodiverticulum, according to the authors’ experience, removal of anterior fixation instruments, gentle repair of the esophageal wall without persistence in definitive and optimal perforation closure, wide local drainage, early enteral nutrition via NGT, and antibiotic prescription is suggested.Before operation, consultation with an experienced neurosurgeon for evaluation of the neck fusion is mandatory. 
